# Supplementary material for: Interactive exploration of biobank-scale ancestral recombination graphs with Lorax
Source: bioRxiv. 2026 Feb 23:2026.02.19.706861. Preprint. [Version 1] doi: 10.64898/2026.02.19.706861 (PMC13160012; doi:10.64898/2026.02.19.706861)
Supplement: Supplement 1 [file media-1.pdf]

Supplementary Fig 1 | Interactive rendering of a large SARS-CoV-2 tree-sequence dataset in Lorax.

Lorax view of local genealogies from the sc2ts dataset, demonstrating real-time rendering and navigation at large scale (~2.4 million sequences). This panel illustrates dense local-tree structure, mutation overlays, and synchronized coordinate-linked visualization under high data volume.

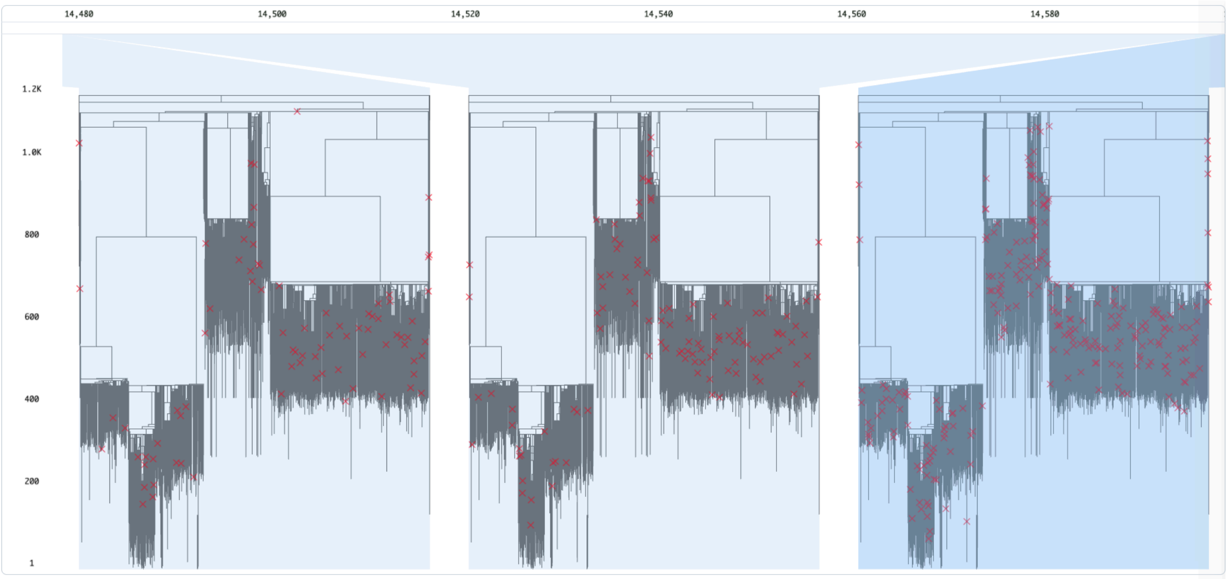

Supplementary Table 1 | Lorax performance across diploid sample size and effective population size (N_e)

Diploid Individuals	N_e	File size (MB)	Local trees	Mutation	Load time (s)	First layout (s)
200,000	5,000	76M	127,802	168,611	1.52	2.77
	50,000	311M	1,263,843	1,683,806	3.75	3.11
600,000	5,000	126M	138,754	182,560	4.00	9.73
	50,000	432M	1,372,235	1,822,947	6.79	10.53
1,000,000	5,000	275M	144,110	188,937	6.56	18.42
	50,000	541M	1,424,671	1,890,836	9.41	16.79
